# Use of Additional Doses of 2024–2025 COVID-19 Vaccine for Adults Aged ≥65 Years and Persons Aged ≥6 Months with Moderate or Severe Immunocompromise: Recommendations of the Advisory Committee on Immunization Practices — United States, 2024

**DOI:** 10.15585/mmwr.mm7349a2

**Published:** 2024-12-12

**Authors:** Lauren E. Roper, Monica Godfrey, Ruth Link-Gelles, Danielle L. Moulia, Christopher A. Taylor, Georgina Peacock, Noel Brewer, Oliver Brooks, George Kuchel, H. Keipp Talbot, Robert Schechter, Katherine E. Fleming-Dutra, Lakshmi Panagiotakopoulos

**Affiliations:** ^1^National Center for Immunization and Respiratory Diseases, CDC; ^2^Gillings School of Global Public Health, University of North Carolina at Chapel Hill, Chapel Hill, North Carolina; ^3^Lineberger Comprehensive Cancer Center, University of North Carolina at Chapel Hill, Chapel Hill, North Carolina; ^4^Watts Healthcare Corporation, Los Angeles, California; ^5^UConn Center on Aging, Claude D. Pepper Older Americans Independence Center, UConn Health, Farmington, Connecticut; ^6^Vanderbilt University, Nashville, Tennessee; ^7^California Department of Public Health.

**Summary**

**What is already known about this topic?**

The Advisory Committee on Immunization Practices (ACIP) recommends 2024–2025 COVID-19 vaccination for all persons aged ≥6 months.

**What is added by this report?**

In October 2024, ACIP recommended that all persons aged ≥65 years and persons aged 6 months–64 years with moderate or severe immunocompromise receive a second 2024–2025 COVID-19 vaccine dose 6 months after their last dose. Further, ACIP recommended that persons aged ≥6 months with moderate or severe immunocompromise may receive additional doses based on shared clinical decision-making.

**What are the implications for public health practice?**

Adults aged ≥65 years should receive 2 doses of 2024–2025 COVID-19 vaccine, and persons aged ≥6 months with moderate or severe immunocompromise should receive ≥2 doses to protect against severe COVID-19.

## Abstract

COVID-19 remains an important cause of morbidity and mortality, especially among adults aged ≥65 years and persons with moderate or severe immunocompromise; these persons are among those at highest risk for severe disease from COVID-19. On June 27, 2024, the Advisory Committee on Immunization Practices (ACIP) recommended 2024–2025 COVID-19 vaccination for all persons aged ≥6 months to target currently circulating strains of SARS-CoV-2 and provide additional protection against severe COVID-19. Because SARS-CoV-2 circulates year-round and immunity from vaccination wanes, on October 23, 2024, ACIP recommended a second 2024–2025 COVID-19 vaccine dose for all adults aged ≥65 years and for persons aged 6 months–64 years with moderate or severe immunocompromise, 6 months after their last dose of 2024–2025 COVID-19 vaccine (minimum interval = 2 months). Further, ACIP recommended that persons aged ≥6 months who are moderately or severely immunocompromised may receive additional doses of 2024–2025 COVID-19 vaccine (i.e., a total of ≥3 doses of 2024–2025 COVID-19 vaccine) based on shared clinical decision-making. Staying up to date with COVID-19 vaccination is recommended to decrease the risk for severe COVID-19, especially among adults aged ≥65 years and persons with moderate or severe immunocompromise.

## Introduction

The overall risk for COVID-19–associated hospitalization and death has decreased in recent years, but COVID-19 continues to cause hundreds of deaths and thousands of hospitalizations in the United States each week (*1*). SARS-CoV-2 circulates year-round with infections and hospitalizations peaking in late summer and winter (*2*). COVID-19–associated hospitalization rates remain higher among adults aged ≥65 years compared with rates among younger adults. During October 2023–August 2024, 70% of COVID-19–associated hospitalizations were among adults aged ≥65 years (*3*). Further, COVID-19 death rates during January 1, 2023–September 30, 2024, were highest among adults aged ≥75 years, followed by rates among adults aged 65–74 years (*2*). Adults aged ≥65 years are less likely to have infection-induced immunity to SARS-CoV-2 compared with adults aged 30–64 years (*2*). In addition, age-related immune system changes result in reduced ability to develop robust immunity after infection or vaccination (*4*,*5*). Thus, older adults are both more reliant on vaccination-related immunity and might require more frequent vaccination for protection against severe illness due to COVID-19. Approximately 6% of persons in the United States have an immunocompromising condition (*6*); however, 16% of persons hospitalized with COVID-19 during July 2023–May 2024 had an immunocompromising condition* (*3*). Persons with moderate or severe immunocompromise might not develop robust immunity after infection or vaccination.

Since June 2020, CDC’s Advisory Committee on Immunization Practices (ACIP) has convened 41 public meetings to review data and consider recommendations related to the use of COVID-19 vaccines (*7*). On June 27, 2024, ACIP recommended that all persons aged ≥6 months receive 2024–2025 COVID-19 vaccination to target currently circulating strains of SARS-CoV-2 and provide additional protection against severe COVID-19–associated illness and death (*8*). In August 2024, the Food and Drug Administration (FDA) approved and authorized the Omicron JN.1 lineage (JN.1 and KP.2 strains) 2024–2025 COVID-19 vaccines by Moderna and Pfizer-BioNTech (KP.2 strain) and Novavax (JN.1 strain) (*9*).

On October 23, 2024, ACIP voted to recommend that, in addition to previously recommended 2024–2025 COVID-19 vaccination, all adults aged ≥65 years and persons aged 6 months–64 years who are moderately or severely immunocompromised (including but not limited to active treatment for malignancy, hematologic malignancies associated with poor responses to COVID-19 vaccines regardless of treatment, solid organ transplant or islet transplant and taking immunosuppressive therapy, chimeric antigen receptor T-cell therapy, hematopoietic cell transplant within 2 years, moderate or severe primary immunodeficiency, advanced or untreated HIV infection, or certain immunosuppressive medications; more details are available at https://www.cdc.gov/vaccines/covid-19/clinical-considerations/interim-considerations-us.html#immunocompromised) should receive a second 2024–2025 COVID-19 vaccine dose. Further, ACIP voted to recommend that persons aged ≥6 months who are moderately or severely immunocompromised may receive additional doses of 2024–2025 COVID-19 vaccine (i.e., a total of ≥3 doses of 2024–2025 COVID-19 vaccine) based on shared clinical decision-making. This report summarizes these ACIP recommendations and the rationale, including evidence reviewed by the ACIP COVID-19 Vaccines Work Group (Work Group) and presented to ACIP.

## Methods

In June 2024, ACIP evaluated published assessments of vaccine effectiveness (VE) and safety of previous COVID-19 vaccine formulations using the Grading of Recommendations, Assessment, Development and Evaluation^†^ approach to guide the recommendations for use of 2024–2025 COVID-19 vaccine in persons aged ≥6 months (*8*). After the June 2024 ACIP meeting, the Evidence to Recommendations Framework (EtR)^§^ was used to evaluate additional data, including VE, vaccine safety, and economic analyses, with a specific focus on information related to additional doses of 2024–2025 COVID-19 vaccine in older adults and persons with immunocompromising conditions.

Since July 2024, the Work Group met seven times to discuss the current policy questions: 1) whether adults aged ≥65 years should receive a second dose of 2024–2025 COVID-19 vaccine, and 2) whether persons aged ≥6 months with moderate or severe immunocompromise should receive ≥1 additional 2024–2025 COVID-19 vaccine doses. The Work Group reviewed evidence on COVID-19 disease surveillance and epidemiology; COVID-19 vaccination coverage, safety, and effectiveness; feasibility of implementation; and cost effectiveness of COVID-19 vaccines (https://www.cdc.gov/acip/evidence-to-recommendations/covid-19-2024-2025-additional-dose.html).

## Rationale and Evidence

### Vaccine Effectiveness

ACIP reviewed CDC data on COVID-19 VE, including data on VE of the original monovalent, bivalent, and 2023–2024 COVID-19 vaccines in the populations of interest. COVID-19 vaccines have provided substantial protection for persons with and without immunocompromise, with generally lower VE in persons with immunocompromise than in those without (*10*). During the 2023–24 respiratory virus season, the 2023–2024 COVID-19 vaccines provided added benefit in a population with a high prevalence of immunity from previous immunization and infection. The 2023–2024 COVID-19 vaccines provided approximately 50% (95% CI = 44%–55%) additional protection against hospitalization initially and then waned to negligible additional protection by approximately 4–6 months after receipt of a 2023–2024 COVID-19 vaccine dose. Protection lasted longer against critical illness (i.e., intensive care unit admission and death). VE against critical illness started at 67% (95% CI = 55%–75%) and decreased to 40% (95% CI = 16%–58%) 4–6 months after the dose, with point estimates indicating additional waning of VE by 6–10 months after the dose (*10*).

Although waning patterns have varied season to season among persons with immunocompromise, an additional COVID-19 vaccine dose has consistently restored protection that has waned after previous doses in persons with and without immunocompromise. During September 2022–August 2023, no residual effectiveness of original monovalent vaccines against COVID-19–associated hospitalization was observed a median of >400 days after the last dose among persons with or without immunocompromise^¶^; receipt of a bivalent dose increased protection to 51% (95% CI = 25%–68%) among persons with immunocompromise and 52% (95% CI = 39%–61%) among persons without immunocompromise (*11*). During September 2023–August 2024, VE of a 2023–2024 COVID-19 vaccine dose among persons with immunocompromise was 36% (95% CI = 22%–48%) 7–59 days after vaccination and 1% (95% CI = −28% to 23%) 120–179 days after vaccination (*9*). In persons without immunocompromise, VE was 51% (95% CI = 45%–56%) 7–59 days after vaccination, waning to 15% (95% CI = 3%–26%) 120–179 days after vaccination (*10*).

### Safety

ACIP reviewed CDC data on COVID-19 vaccine safety with a focus on doses administered after the initial vaccination series. Robust safety surveillance of COVID-19 vaccines has demonstrated that serious adverse events are rare: anaphylactic reactions have been rarely reported after receipt of COVID-19 vaccines (*12*), and a rare risk for myocarditis and pericarditis has been observed after COVID-19 vaccination, predominantly among males aged 12–39 years (*13*). No increased risk for myocarditis or pericarditis was observed in adults aged ≥65 years after COVID-19 vaccination (*13*); whether the risk might be different in persons with immunocompromise is unknown.

COVID-19 vaccine doses are reactogenic (*2*). Compared with doses in the initial vaccination series, the rate of local and systemic reactions reported to V-safe, a voluntary smartphone-based U.S. safety surveillance system established by CDC to monitor health after COVID-19 vaccination, was lower after subsequent doses (*14*,*15*). Most vaccine recipients have mild reactions; however, during 2023–2024, ≥10% of COVID-19 vaccine recipients in V-safe reported health impact events during the 7 days after vaccination, such as being unable to complete daily activities (*16*). Reactogenicity observed in V-safe after a bivalent COVID-19 vaccine dose was milder and less frequent among older adults compared with adolescents and younger adults (*17*).

### Economic Analyses

ACIP considered whether a second dose of 2024–2025 COVID-19 vaccine in persons aged ≥65 years and persons aged ≥6 months with moderate or severe immunocompromise is a reasonable and efficient allocation of resources. The societal incremental cost-effectiveness ratio (ICER) for an additional dose of COVID-19 vaccine in persons aged ≥65 years was $356,534 per quality-adjusted life year saved for the base case estimate (*18*). ICER values were sensitive to seasonality-adjusted vaccine impact, probability of hospitalization, and vaccine cost. Estimates of ICER values that approximate cost effectiveness for those with higher risk for COVID-19–associated hospitalization, such as persons with underlying conditions, were more favorable (*18*). Data were not available specific to cost-effectiveness in persons with moderate or severe immunocompromise.

## Updated Recommendations for 2024–2025 COVID-19 Vaccination for Persons Aged ≥65 Years

On October 23, 2024, ACIP recommended that all persons aged ≥65 years** receive a second dose of 2024–2025 COVID-19 vaccine (Table) 6 months^††^ (minimum interval = 2 months) after the last dose of 2024–2025 COVID-19 vaccine. If an adult aged ≥65 years is previously unvaccinated and receiving Novavax, 2 doses are recommended as an initial vaccination series and should be followed by a third dose of any age-appropriate 2024–2025 COVID-19 vaccine 6 months (minimum interval = 2 months) after the second dose.

**TABLE:** Routine 2024–2025 COVID-19 vaccination schedule for persons aged ≥65 years,* by COVID-19 vaccination history^†^ — United States, October 2024

COVID-19 vaccination history before 2024–2025 vaccine	No. of 2024–2025 COVID-19 doses recommended	2024–2025 vaccination schedule
≥1 mRNA vaccine dose (Moderna or Pfizer-BioNTech) or ≥2 Novavax doses or ≥1 Janssen dose	2	2024–2025 dose 1 (Moderna, Novavax, or Pfizer-BioNTech): ≥8 wks after last dose 2024–2025 dose 2 (Moderna, Novavax, or Pfizer-BioNTech): 6 mos (minimum interval = 2 mos) after 2024–2025 dose 1
1 Novavax dose	2	2024–2025 dose 1 (Novavax): 3–8 wks after last dose^§^ 2024–2025 dose 2 (Moderna, Novavax, or Pfizer-BioNTech): 6 mos (minimum interval = 2 mos) after 2024–2025 dose 1
Unvaccinated	2	2024–2025 dose 1 (Moderna or Pfizer-BioNTech): day 0 2024–2025 dose 2 (Moderna, Novavax, or Pfizer-BioNTech): 6 mos (minimum interval = 2 mos) after dose 1 or
	3	2024–2025 dose 1 (Novavax): day 0 2024–2025 dose 2 (Novavax): 3–8 wks after dose 1^§^ 2024–2025 dose 3 (Moderna, Novavax, or Pfizer-BioNTech): 6 mos (minimum interval = 2 mos) after dose 2

* Routine schedule applies to persons who are not moderately or severely immunocompromised. Additional clinical considerations, including detailed schedules and tables by age and vaccination history, are available at https://www.cdc.gov/vaccines/covid-19/clinical-considerations/covid-19-vaccines-us.html.

^†^ COVID-19 vaccination history refers to all doses of COVID-19 vaccine from any manufacturer received before the availability of the 2024–2025 COVID-19 vaccines and includes original, bivalent, and 2023–2024 COVID-19 vaccines.

^§^ If ≥8 weeks have elapsed since receipt of the first dose of Novavax, any 2024–2025 COVID-19 vaccine (i.e., Moderna, Novavax, or Pfizer-BioNTech) may be administered.

## Updated Recommendations for 2024–2025 COVID-19 Vaccination for Persons Aged ≥6 Months with Moderate or Severe Immunocompromise

On October 23, 2024, ACIP recommended that persons aged 6 months–64 years with moderate or severe immunocompromise^§§^ receive a second dose of 2024–2025 COVID-19 vaccine 6 months^¶¶^ after the last 2024–2025 COVID-19 vaccine dose (minimum interval = 2 months). For all persons with moderate or severe immunocompromise, ≥2 doses of 2024–2025 COVID-19 vaccine are recommended; 1 of the 2 recommended 2024–2025 COVID-19 vaccine doses may be a part of the initial vaccination series, and in this case, the remaining dose is recommended 6 months (minimum interval = 2 months) after completion of the initial vaccination series.​ ACIP also recommended that persons aged ≥6 months with moderate or severe immunocompromise may receive additional 2024–2025 COVID-19 vaccine doses (i.e., a total of ≥3 doses of 2024–2025 COVID-19 vaccine) based on shared clinical decision-making,*** which should be guided by the clinical judgment of a health care provider and personal preference and circumstances of the patient (*18*). Additional clinical considerations, including detailed schedules and tables by age and vaccination history for persons with and without moderate or severe immunocompromise, are available at https://www.cdc.gov/vaccines/covid-19/clinical-considerations/covid-19-vaccines-us.html.

### Implementation Considerations

These recommendations were based on persistent SARS-CoV-2 circulation throughout the year, higher risk for severe illness attributable to COVID-19 in adults aged ≥65 years and persons with moderate or severe immunocompromise, protection anticipated from the updated vaccines against JN.1 and other closely related circulating SARS-CoV-2 variants, the expected waning of COVID-19 VE, and additional implementation considerations, including facilitating clear communication and equitable access to vaccination (*2*). The available data indicate that persons aged ≥65 years and those with moderate or severe immunocompromise should receive 2 doses of 2024–2025 COVID-19 vaccine, at an interval of 6 months, to enhance protection throughout the year. However, although the recommended interval between these doses is 6 months, the minimum interval of 2 months allows for flexibility of vaccine administration when accounting for individual risk and circumstances.

Persons aged ≥6 months with moderate or severe immunocompromise may receive additional 2024–2025 COVID-19 vaccine doses based on shared clinical decision-making. Vaccine recommendations using shared clinical decision-making are individually based and guided by a discussion between the health care provider and the patient or their parent or guardian (*19*). Although shared clinical decision-making recommendations can be difficult to implement (*20*), this recommendation allows persons with moderate or severe immunocompromise to time additional doses of COVID-19 vaccine around immunosuppressive treatments, after which recipients might be at increased risk of severe COVID-19, or around travel and other life events, during which they might have increased risk of exposure to SARS-CoV-2 (*2*).

Persons can self-attest to their moderately or severely immunocompromised status and receive COVID-19 vaccine doses wherever vaccines are offered. Vaccinators should not deny COVID-19 vaccination to a person because of a lack of documentation of their immunocompromised status. A description of immunocompromising conditions and considerations is available at https://www.cdc.gov/vaccines/covid-19/clinical-considerations/interim-considerations-us.html#immunocompromised.

### Reporting Vaccine Adverse Events

Adverse events after vaccination should be reported to the Vaccine Adverse Event Reporting System (VAERS). For licensed COVID-19 vaccines administered to persons aged ≥12 years, reporting is encouraged for any clinically significant adverse event, even when a causal association between the vaccine and the event is uncertain, as well as for vaccine administration errors. For COVID-19 vaccines given under Emergency Use Authorization,^†††^ vaccination providers are required to report certain adverse events to VAERS. Additional information is available at https://vaers.hhs.gov or by telephone at 1-800-822-7967.

## Footnotes

* Immunocompromising conditions included AIDS or CD4 count <200; complement deficiency; graft versus host disease; HIV infection; immunoglobulin deficiency; immunosuppressive therapy ≤12 months before admission; leukemia, lymphoma, solid organ malignancy, metastatic cancer, or multiple myeloma diagnosed ≤12 months of admission or currently in treatment; bone marrow transplant; steroid therapy (≤2 weeks before admission; excluding inhaled, intranasal steroids or intramuscular or intra-articular injection of steroids); solid organ transplant; or other conditions typically associated with immunocompromised status upon review.

† https://www.cdc.gov/acip/evidence-based-recommendations/

§ Through the EtR, the Work Group reviewed data on the public health problem of COVID-19 among older adults and persons with immunocompromising conditions, as well as the benefits and harms, value to the target population, acceptability to key stakeholders, feasibility, societal resource use, and equity implications of additional doses of COVID-19 vaccines.

¶ Effectiveness of original monovalent vaccine against COVID-19–associated hospitalization a median of >400 days after the last dose was 14% (95% CI = −9% to 33%) among persons with immunocompromise and 6% (95% CI = −7% to 17%) among persons without immunocompromise.

** ACIP voted (15 to zero) to recommend a second dose of 2024–2025 COVID-19 vaccine for persons aged ≥65 years.

†† For adults aged ≥65 years, the recommended interval of the second dose is 6 months but can be as early as 2 months or later than 6 months after the first dose of 2024–2025 COVID-19 vaccine.

§§ ACIP voted (15 to zero) to recommend a second dose of 2024–2025 COVID-19 vaccine for persons aged 6 months–64 years with moderate or severe immunocompromise.

¶¶ For persons aged ≥6 months who are immunocompromised, the recommended interval of the second dose is 6 months but can be as early as 2 months or later than 6 months after the first dose of 2024–2025 COVID-19 vaccine.

*** ACIP voted (15 to zero) to recommend additional doses of 2024–2025 COVID-19 vaccine for persons aged ≥6 months with moderate or severe immunocompromise (minimum interval = 2 months) under shared clinical decision-making.

††† 2024–2025 COVID-19 vaccines under FDA Emergency Use Authorization are Moderna and Pfizer-BioNTech for use among persons aged 6 months–11 years and Novavax for use among persons aged ≥12 years.
